# ﻿The complete mitochondrial DNA sequences of two sibling species of lumbricid earthworms, *Eiseniafetida* (Savigny, 1826) and *Eiseniaandrei* (Bouché, 1972) (Annelida, Crassiclitellata): comparison of mitogenomes and phylogenetic positioning

**DOI:** 10.3897/zookeys.1097.80216

**Published:** 2022-04-29

**Authors:** Csaba Csuzdi, Jachoon Koo, Yong Hong

**Affiliations:** 1 Department of Zoology, Eszterházy Károly Catholic University, Eger, Hungary; 2 Division of Science Education and Institute of Fusion Science, College of Education, Jeonbuk National University, Jeonju 54896, Republic of Korea; 3 Department of Agricultural Biology, College of Agriculture & Life Science, Jeonbuk National University, Jeonju 54896, Republic of Korea

**Keywords:** Compost worms, mitogenome, Oligochaeta, phylogeny, sibling species

## Abstract

Composting earthworms of the genus *Eisenia* play an important role in soil ecosystems. However, taxonomic classification of this genus, especially the sibling species *Eiseniafetida* and *Eiseniaandrei*, is complicated because of their morphological similarity. In this study, we assessed the utility of the complete mitochondrial genome (mitogenome) for identification and differentiation of the two species. The complete mitogenomes of *E.andrei* and *E.fetida* were 15,714 and 16,560 bp, respectively. They contained 37 genes, comprising 13 protein-coding genes (PCGs), two rRNA genes, 22 tRNA genes, and a putative non-coding region, as observed in other earthworms. Sequence comparisons based on the complete nucleotide sequences excluding the non-coding region showed 85.8% similarity, whereas the predicted amino acid sequences of the 13 PCGs were 92.7% similar between the two species. In particular, distinct features were found in the non-coding regions of the mitogenomes. They include a control region associated with putative mitogenome replication and an extended sequence. The extended sequence showed significant differences between the two species and other known earthworm species, suggesting its potential as a feasible molecular marker for species identification. Phylogenetic analysis of the 36 mitogenomes of earthworm species corroborated the monophyly of the genus *Eisenia* and the taxonomic distinctness of the sibling species pair, *E.fetida* and *E.andrei*.

## ﻿Introduction

The earthworm species *Eiseniafetida* was described as *Enterionfetidum* by Savigny (1826). Eisen (1873) relegated this species to his newly described genus *Allolobophora* Eisen, 1873 and remarked that it is easily recognized by its peculiar color pattern consisting of reddish-brown bands separated by yellowish intersegments. Later, Malm (1877) selected *Enterionfetidum* as the type species of the genus *Eisenia* Malm, 1877. For a long time, the characteristic striated pattern was a primary identifiable characteristic of the species until Avel (1937) recognized that the classical *Eiseniafetida* existed in two morphological variants: a typical striped form and an evenly pigmented form that might represent a separate species (Avel 1937).

André (1963) carried out breeding experiments with earthworms and recognized that reproductive isolation exists between the striped and evenly colored forms of *E.fetida*, and that the crossbred offspring are sterile. Consequently, he described the uniformly pigmented form as Eiseniafetidavar.unicolor. Variety names proposed after 1961 were considered intrasubspecific and invalid; therefore, Bouché (1972) proposed a new name for var. unicolor, *Eiseniafetidaandrei* Bouché, 1972. Since then, various authors have treated the subspecies *E.f.andrei* differently. Reynolds (1977), Sims (1983), Easton (1983), Csuzdi and Zicsi (2003), and Blakemore (2008, 2013) regarded it as a color morph and synonym of *Eiseniafetida* (Savigny, 1826). Others, such as Sims and Gerard (1985), Qiu and Bouché (1998), Lehmitz et al. (2014), and Martin et al. (2016), considered *E.fetida* and *E.andrei* to be two distinct valid species.

*Eiseniafetida* is an important composting worm and ecotoxicological test organism (Domínguez et al. 2005; Römbke et al. 2016). Therefore, intensive studies have been carried out since the early 1980s to determine whether the two types of *E.fetida* (striped and unicolor) represent two morphological variants or two separate species. The first clear indication that *E.fetida* and *E.andrei* might represent two separate species was presented by Jaenike (1982) who used an electrophoretic survey to demonstrate complete reproductive isolation between the two species. Later, Reinecke and Viljoen (1991) and Domínguez et al. (2005), using crossbreed experiments, reported complete reproductive isolation between the two species (no viable cocoons were observed in interspecific crosses) and noted that the two species differed in their life histories. Furthermore, *E.andrei* exhibited higher reproduction rates.

Recently, Römbke et al. (2016) carried out a detailed barcoding study of the *Eiseniafetida* / *E.andrei* complex using samples from 28 laboratories in 15 countries. The two species formed two distinct clades on the neighbor-joining tree, and the *E.fetida* clade consisted of two subclades, *fetida* 1 and *fetida* 2. The mean uncorrected p-distances were 14.2% between *fetida*1 and *andrei*, 14.3% between *fetida*2 and *andrei*, and 11.2% between the two *fetida* subclades; these values exceed the species-level threshold suggested by Chang and James (2011). Therefore, Römbke et al. (2016) concluded that the complex consists of three taxa: *E.andrei* and two cryptic taxa, *E.fetida* 1 and *E.fetida* 2. Moreover, they found that *E.andrei* was always correctly identified from its morphology, whereas *E.fetida* was often misidentified as *E.andrei* (Römbke et al. 2016).

It is worth mentioning that the native range of *E.fetida* and *E.andrei* is unknown. All of the above-mentioned studies were based on laboratory stocks or specimens collected from compost or manure heaps. Perel (1998) hypothesized that the native range of *E.fetida* is somewhere in the forest-steppe zone of Central Asia; therefore, Latif et al. (2017) barcoded 62 new specimens of this complex collected from different anthropogenic and natural habitats in Iran. Surprisingly, all Iranian material appeared in the *E.andrei* clade, irrespective of striped or uniform pigmentation. Moreover, the *E.andrei* clade showed high genetic structuring in contrast to the almost uniform genetic composition found by Römbke et al. (2016). Automatic barcode gap discovery (ABGD) analysis identified two species corresponding to *Eiseniaandrei* and *Eiseniafetida* with high genetic structuring inside both species, but neither of the subclades reached the unambiguous species threshold [15% K2P distance according to Chang and James (2011)].

Comparison of mitogenomes may reveal important genome-level characteristics, helping us understand genome structure, gene order, phylogenetic relationships, and evolutionary lineages. The earthworm mitogenome is a circular, double-stranded, covalently closed DNA molecule containing 13 protein-coding genes (PCGs), two ribosomal RNA genes (rRNAs), 22 transfer RNA genes (tRNAs), and one non-coding region (Zhang et al. 2016). Although Lumbricidae is the most important earthworm family in the Northern Hemisphere temperate zone and contains many widespread and invasive cosmopolitan species, only a few complete or nearly complete mitogenomes are available for this family (Boore and Brown 1995; Shekhovtsov and Peltek 2019; Zhang et al. 2019; Shekhovtsov et al. 2020). In the present study, we sequenced the complete mitochondrial genome of the sibling species *E.fetida* and *E.andrei* to clarify its taxonomic position and to gain a better understanding of the mitogenomes of Lumbricidae.

## ﻿Material and methods

### ﻿Sample preparation and DNA extraction

Adult *E.andrei* were collected from a farm in Sangseo-myeon, Buan-gun, Jeollabuk-do, Korea (33°41'23.80"N, 126°38'33.67"E; 40 m a.s.l.) on March 26, 2021. *Eiseniafetida* adults were collected near a house at Seolcheon-myeon, Muju-gun, Jeollabuk-do, Korea (33°58'00.61"N, 127°47'47.88"E; 408 m a.s.l.) on April 2, 2021, and preserved in 99% ethanol until DNA extraction. A voucher specimen of each species was deposited at Jeonbuk National University, Jeonju City, Korea, under accession numbers JBNU0011 and JBNU0012. Total genomic DNA was prepared from a small portion of body segments of a single adult earthworm using the QIAamp DNA Mini Kit (Qiagen, Hilden, Germany). The remaining tissue was stored at -20 °C in 90% ethanol to preserve the specimens.

### ﻿TruSeq DNA Library construction

The sequencing library was prepared by random fragmentation of genomic DNA, followed by 5’ and 3’ adapter ligations. Briefly, 100 ng genomic DNA was fragmented using adaptive focused acoustic (AFA) technology (Covaris Inc., Woburn, MA, USA). The fragmented DNA was end-repaired and ligated to TruSeq indexing adapters using the Illumina TruSeq DNA Nano Library Prep Kit according to the manufacturer’s instructions (Illumina Inc., San Diego, CA, USA). The resulting libraries were quantified through a qPCR-based assay using the KAPA Library Quantification Kit for Illumina Sequencing platforms according to the manufacturer’s instructions (Kapa Biosystems, Woburn, MA, USA). The libraries were qualified using an Agilent Technologies 2200 TapeStation (Agilent Technologies, Santa Clara, CA, USA).

### ﻿DNA sequencing and assembly

Paired-end (2 × 150 bp) sequencing was performed using an Illumina HiSeq-X platform (Illumina Inc., USA) at Macrogen Inc. (Seoul, Korea). For each species, > 39 million reads (5.1–5.9 Gb) were generated. To reduce bias in the analysis, adapter trimming and quality filtering were performed using Trimmomatic version 0.36 (Bolger et al. 2014). After filtering, the number of total reads of *E.andrei* and *E.fetida* was > 29 million (4.4 Gb) and > 34 million (5.2 Gb), respectively. De novo assembly of raw sequencing reads was performed using various *k*-mer lengths in SPAdes version 3.13.0 (Bankevich et al. 2012). Mitochondrial contigs were assembled into a single contig using BlastN alignment (https://blast.ncbi.nlm.nih.gov/Blast.cgi) against the *Lumbricusterrestris* Linnaeus, 1758 mitogenome (GenBank accession number, NC_001673) as the reference sequence. The assembled mitochondrial sequences for *E.andrei* were connected to a single circular molecule, whereas the conformation of the contig for *E.fetida* was unclear because the 12 bp TA-repeat sequence overlapped at both ends. This region corresponds to heteroplasmic tandem repeats in the mitochondrial control region (Liu et al. 2020). To close the circular genome, pairs of PCR primers (5’-ACCACCAGAGTTCTCGTTCG-3’ and 5’-GCCAATATCGGCCCAAAACC-3’) were designed to amplify the control region. The reaction was performed in an nTaq-tenuto (Enzynomics Inc., Seoul, Korea) with the following program: 95 °C for 3 min; 35 cycles of 95 °C for 20 s, 55 °C for 30 s, and 72 °C for 1 min; and a final extension of 5 min at 72 °C. The amplicons were directly sequenced using Sanger sequencing (Macrogen Inc., Seoul, Korea) to determine the complete mitogenome of *E.fetida*.

### ﻿Mitogenome annotation

The annotation and visualization of mitochondrial genomes were performed using the online MITOS software (Donath et al. 2019), and manual curation was performed using BLAST searches in the NCBI database for various earthworm mitochondrial genomes deposited in NCBI (Table 1). A comparative map of mitochondrial genomes was created using Geneious Prime 2021 software (https://www.geneious.com). The *cox1* sequence was used as an anchor for linearized maps of the mitochondrial genomes. The annotated complete genome sequences were registered in GenBank under accession numbers OK513069 for *E.andrei* and OK513070 for *E.fetida*. The associated biosample numbers were SAMN26185682 for *E.andrei* and SAMN26185683 for *E.fetida*. All sequencing datasets, including SRA, are available in the NCBI BioProject database under the accession number PRJNA769829.

**Table 1.** List of Megadrili mitogenomes used in this study.

Species	Genbank No.	Total length (bp)	*Non-coding region (bp)	Topology
*Amynthasaspergillus*	KJ830749	15,115	565	Circular
*Amynthascarnosus*	KT429008	15,160	601	Circular
*Amynthascorticis*	KM199290	15,126	573	Circular
*Amynthascucullatus*	KT429012	15,122	569	Circular
*Amynthasgracilis*	KP688582	15,161	582	Circular
*Amynthashupeiensis*	KT429009	15,069	477	Circular
*Amynthasinstabilis*	KT429007	15,159	577	Circular
*Amynthasjiriensis*	KT783537	15,151	618	Circular
*Amynthaslongisiphonus*	KM199289	15,176	491	Circular
*Amynthasmoniliatus*	KT429020	15,133	562	Circular
*Amynthaspectiniferus*	KT429018	15,188	618	Circular
*Amynthasredactus*	KT429010	15,131	572	Circular
*Amynthasrobustus*	KT429019	15,013	432	Circular
*Amynthasrongshuiensis*	KT429014	15,086	546	Circular
*Amynthasspatiosus*	KT429013	15,152	595	Circular
*Amynthastriastriatus*	KT429016	15,160	582	Circular
*Amynthasyunoshimensis*	LC573969	15,109	581	Circular
*Metaphirecalifornica*	KP688581	15,147	567	Circular
*Metaphireguillelmi*	KT429017	15,174	594	Circular
*Metaphirehilgendorfi*	LC573968	15,186	649	Circular
*Metaphirevulgaris*	KJ137279	15,061	484	Circular
*Duplodicodrilusschmardae*	KT429015	15,156	595	Circular
*Perionyxexcavatus*	EF494507	15,083	504	Circular
*Tonoscolexbirmanicus*	KF425518	15,170	595	Circular
*Aporrectodearosea*	MK573632	15,086	512	Circular
*Lumbricusrubellus*	MN102127	15,464	433	Circular
*Lumbricusterrestris*	U24570	14,998	384	Circular
**Eiseniabalatonica	MK642872	14,589	-	Linear
**Eisenianana	MK618511	14,599	-	Linear
**Eisenianordenskioldi	MK618509	14,572	-	Linear
**Eisenianordenskioldi	MK618510	14,592	-	Linear
**Eisenianordenskioldi	MK618513	14,567	-	Linear
**Eisenianordenskioldi	MK642867	14,576	-	Linear
**Eisenianordenskioldi	MK642868	14,556	-	Linear
**Eisenianordenskioldipallida	MK618512	14,567	-	Linear
**Eisenianordenskioldipallida	MK642869	14,553	-	Linear
**Eiseniaspelaea	MK642870	14,738	-	Linear
**Eiseniatracta	MK642871	14,589	-	Linear
*Eiseniaandrei*	OK513069	15,714	1151	Circular
*Eiseniafetida*	OK513070	16,560	1988	Circular
*Drawidajaponica*	KM199288	14,648	3	Circular
*Pontoscolexcorethrurus*	KT988053	14,835	318	Circular

*Putative non-coding region between *trnR* and *trnH*. ** Incomplete mitochondrial genome sequence lacking the entire non-coding region and *trnR*.

### ﻿Phylogenetic analyses

To clarify the phylogenetic position of the two species, the available complete or near-complete mitogenomes were obtained from GenBank, comprising 24 species of Megascolecidae, 14 species of Lumbricidae, and one species of Rhinodrilidae. *Drawidajaponica* (Michaelsen, 1892) from the exquisiclitellate family Moniligastridae was used as the outgroup.

Two sets of sequence matrices were composed: one containing the PCGs, 12S, and 16S RNA genes, and the other consisting only of PCGs. Sequences were aligned with MAFFT ver. 7 (Katoh and Standley 2013) using the G-INS-i option and concatenated in MegaX (Kumar et al. 2018); the resulting matrices were 13,505 and 11,241 bp, respectively. The protein-coding alignment was translated into amino acid sequences and aligned in MAFFT ver. 7 using the G-INS-i option; the resulting matrix was with 3714 amino acid positions.

The best-fitting evolutionary model for each partition (PCG, 16S, 12S) was selected using ModelFinder (Kalyaanamoorthy et al. 2017) implemented in the IQTree web server (http://iqtree.cibiv.univie.ac.at/) by applying the Akaike information criterion (AIC; Akaike 1973) and Bayesian information criterion (BIC; Schwarz 1978). GTR + I + Γ was selected as the best-fitting evolutionary model for PCGs and 12S RNA, TIM2 I + Γ was selected for 16S RNA, and MtMAM I + Γ for the amino acid sequences.

Bayesian inference of the phylogeny was estimated with MrBayes v.3.2.6 (Ronquist et al. 2012) as implemented in CIPRES Science Gateway V. 3.3. (Miller et al. 2010). The analysis was performed with default parameters, and each of the two independent runs was set to 10 million generations and sampling every 1000^th^ generation (10,000 trees). Twenty percent of the trees were discarded as burn-in, and the remaining trees were combined and summarized in a 50% majority-rule consensus tree. As the TIM2 model was not implemented in MrBayes, the closest complex model GTR + I + Γ was used instead. Maximum likelihood phylogenetic inference was performed using the IQTree web server with default options (Nguyen et al. 2015http://iqtree.cibiv.univie.ac.at/).

## ﻿Results

**Figure 1.**
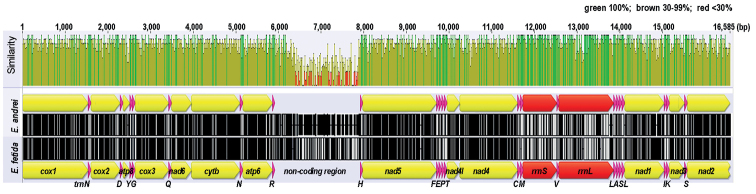
Comparison of mitogenomes of *Eiseniaandrei* and *E.fetida*. The map is based on sequence similarity and was constructed using Geneious Prime 2021 software. Sequence similarity is represented by green (100%), brown (30–99%), and red (<30%). *cox1* was used as an anchor to linearized genomes. Organization of mitochondrial genes is shown in Table 2. Non-coding region is defined as the region between *trnR* and *trnH*.

The complete mitochondrial genomes of *Eiseniafetida* and *Eiseniaandrei* consisted of 16,560 and 15,714 base pairs, respectively. The setup of the mitogenomes of both species followed the typical Bauplan of the earthworm mitogenome assembly, consisting of 13 PCGs, 22 transfer RNAs, two ribosomal RNA genes, and a control region (Fig. 1; Table 2).

**Table 2.** Comparative analysis of gene organization of *Eiseniaandrei* and *E.fetida* mitogenomes (bp = base pairs).

Gene	Strand	*E.fetida*		*E.andrei*		Similarity
		Size (bp)	start/stop codon	Size (bp)	start/stop codon	
*cox1*	+	1540	ATG/T	1540	ATG/T	86%
*trnN*	+	61		61		98%
*cox2*	+	687	ATG/TAG	687	ATG/TAA	86%
*trnD*	+	61		61		89%
*atp8*	+	163	ATG/T	160	ATG/T	78%
*trnY*	+	63		63		95%
*trnG*	+	63		64		92%
*cox3*	+	778	ATG/T	778	ATG/T	86%
*trnQ*	+	69		69		91%
*nad6*	+	469	ATG/T	469	ATG/T	85%
*cytb*	+	1140	ATG/TAA	1140	ATG/TAA	85%
*trnW*	+	62		63		90%
*atp6*	+	696	ATG/TAA	696	ATG/TAA	82%
*trnR*	+	61		63		93%
*NC	+	1988		1151		60%
*trnH*	+	62		62		90%
*nad5*	+	1722	ATG/TAA	1722	ATG/TAA	83%
*trnF*	+	62		63		92%
*trnE*	+	63		63		95%
*trnP*	+	64		64		94%
*trnT*	+	65		63	-	97%
*nad4L*	+	297	ATG/TAA	297	ATG/TAA	88%
*nad4*	+	1359	ATG/TAG	1359	ATG/TAG	83%
*trnC*	+	65		65		97%
*trnM*	+	63		63		100%
*rrnS*	+	794		794		94%
*trnV*	+	64		63		94%
*rrnL*	+	1282		1278		89%
*trnL*	+	62		63		94%
*trnA*	+	62		62		94%
*trnS*	+	67		67		94%
*trnL*	+	64		62		95%
*nad1*	+	919	ATG/T	919	ATG/T	85%
*trnI*	+	64		64		97%
*trnK*	+	65		65		97%
*nad3*	+	354	ATG/TAG	354	ATG/TAG	82%
*trnS*	+	64		64		97%
*nad2*	+	1003	ATG/T	1003	ATG/T	81%

* Non-coding regions between *trnR* and *trnH*.

All genes were encoded on the heavy DNA strand, and both genomes showed biased base composition, with 63.5% AT and 36.4% GC content in *E.fetida* and 62.8% and 37.2% in *E.andrei*.

The overall mitogenome sequence similarity between the two species was 80.8%, and it increased to 85.8% when the control region was excluded. The 13 PCGs were 78%–86% similar (Table 2). Among the PCGs, *nad4l* showed the highest similarity (88%) and *atp8* the lowest (78%). The average similarity of the 13 PCGs between the two species was 84%.

However, the deduced amino acid sequences of the 13 PCGs showed, on average, 92.7% similarity between the species; COX1 was the most similar (99.4%) and ATP8 the most dissimilar (79.6%) (Table 3). Sequence variation between the two species was lower at the amino acid level than at the DNA level. In particular, *cox1* showed 86% similarity at the DNA level but more than 99% similarity at the amino acid level.

**Table 3.** Comparison of deduced amino acid sequences of 13 protein-coding genes between *Eiseniaandrei* and *E.fetida*.

Protein	*Eiseniafetida*	*Eiseniaandrei*	Similarity (%)
cox1	513 aa	513 aa	99.4
cox2	228 aa	228 aa	95.2
atp8	54 aa	53 aa	79.6
cox3	259 aa	259 aa	97.7
nad6	156 aa	156 aa	93.6
cytb	379 aa	379 aa	96.0
atp6	231 aa	231 aa	93.1
nad5	567 aa	573 aa	90.8
nad4l	98 aa	98 aa	92.9
nad4	452 aa	452 aa	92.0
nad1	306 aa	306 aa	92.6
nad3	117 aa	117 aa	92.3
nad2	334 aa	334 aa	89.1

**Figure 2.**
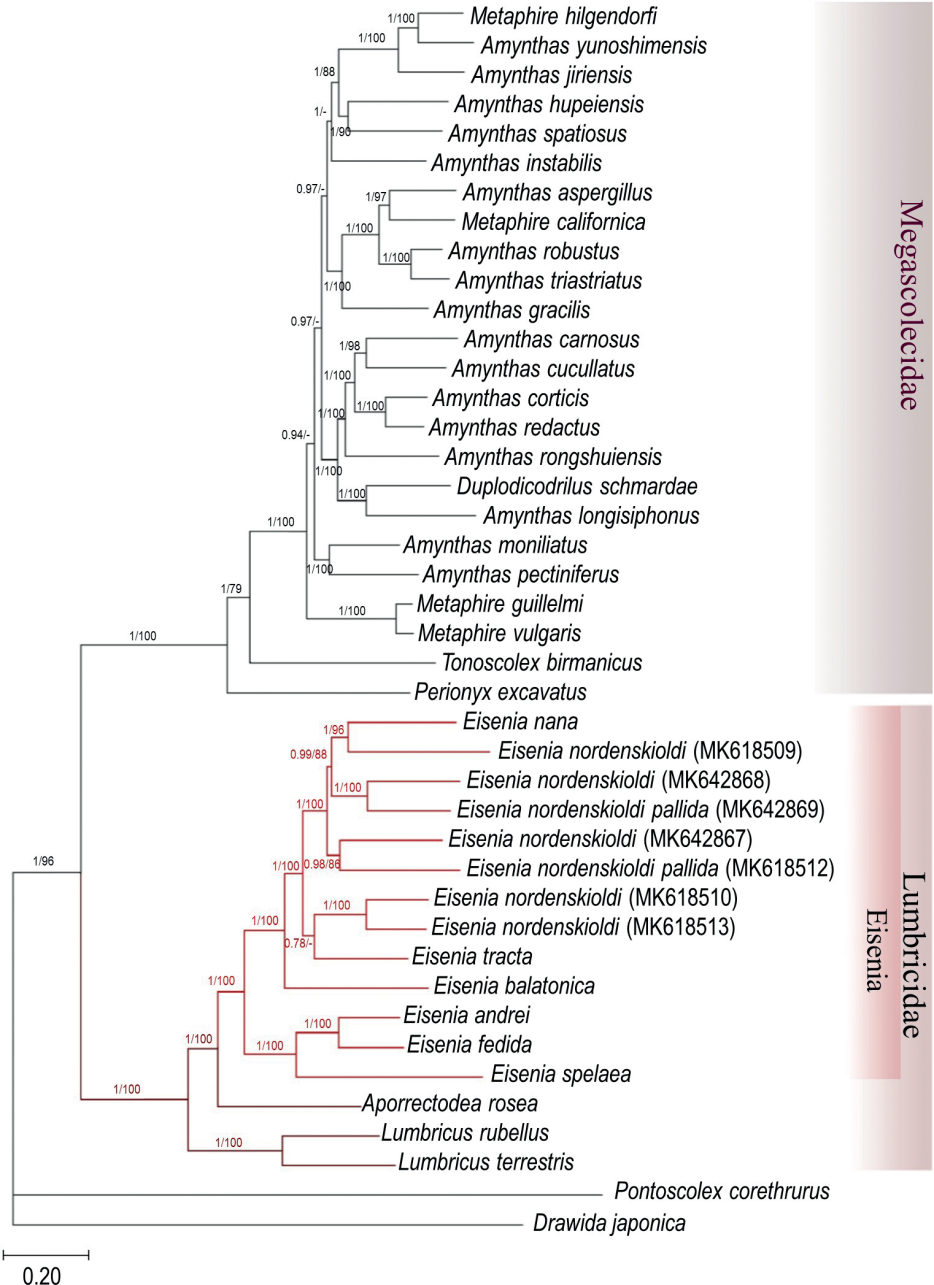
Phylogenetic analysis of 42 Megadrili species, including *E.andrei* and *E.fetida*, based on nucleotide sequences of 13 protein-coding genes and the 12S and 16S RNA genes. The numbers above branches present Bayesian posterior probabilities/maximum likelihood bootstrap values (values under 0.75 and 75% are not shown).

**Figure 3.**
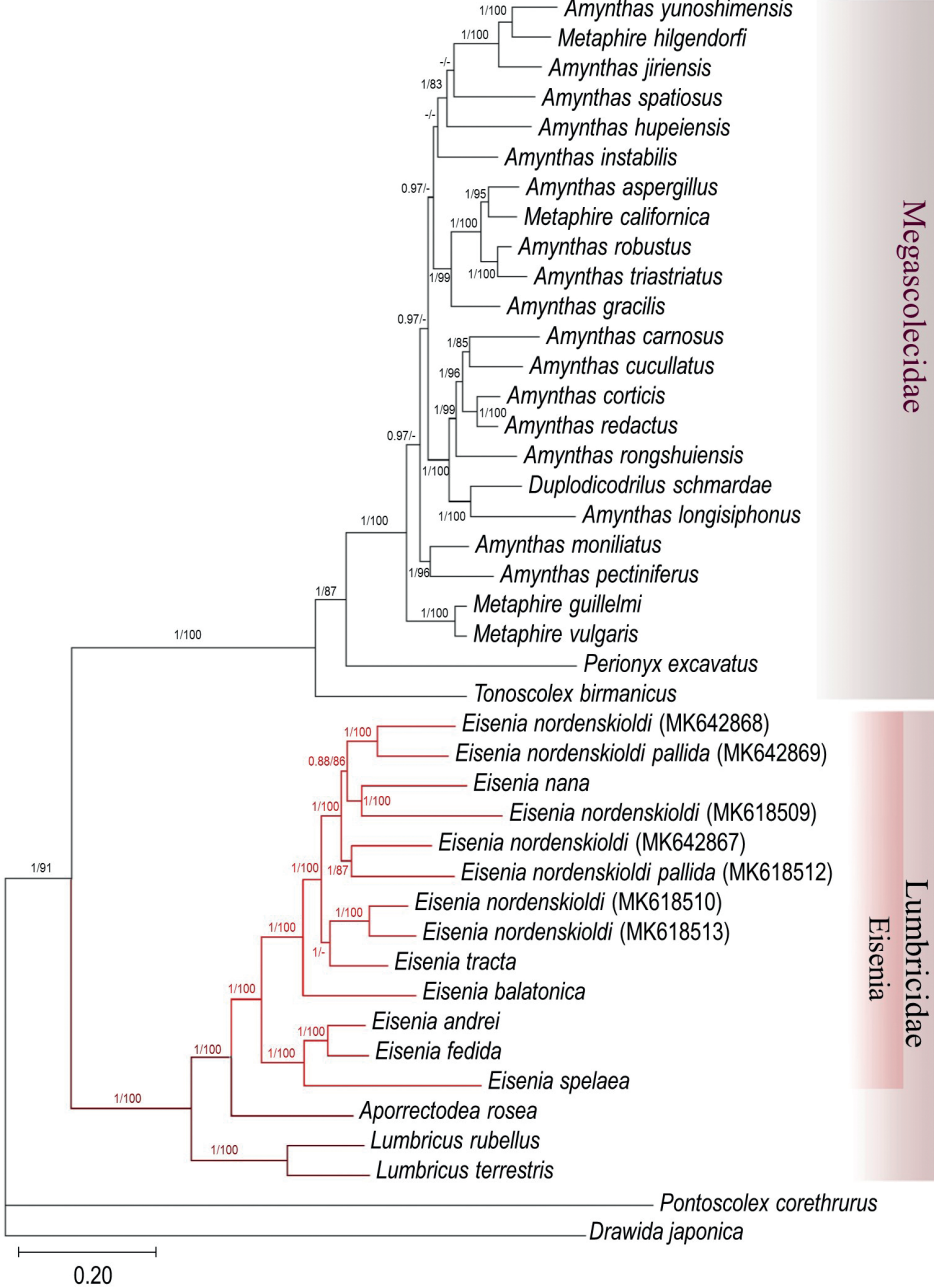
Phylogenetic analysis of 42 Megadrili species, including *E.andrei* and *E.fetida*, based on translated amino acid of 13 protein-coding genes. The numbers above branches are Bayesian posterior probabilities/maximum likelihood bootstrap values (values under 0.75 and 75% are not shown).

Phylogenetic reconstruction of the available Lumbricidae complete or nearly complete mitogenomes using the 13 PCGs and the 12S and 16S RNA genes highly supported the Lumbricidae family (1 posterior probability and 100% bootstrap support). In addition, the genus *Eisenia* was resolved monophyletic, and the close relationship of the *E.fetida/andrei* species pairs was confirmed (Fig. 2). Interestingly, the included *Eisenia* sequences formed two well-supported subclades: one consisting of the European *E.spelaea* (Rosa, 1901) and the *E.fetida*/*andrei* species pair, and the other comprising the Asian taxa of the *E.nordenskioldi* (Eisen, 1879) species complex (including *E.tracta* Perel, 1985 and *E.nana* Perel, 1985), and the Asian specimens of the European *E.balatonica* (Pop, 1943). A nearly identical tree topology was obtained using the translated amino acid sequences. The only notable difference was in the swapped position of *Perionyxexcavatus* Perrier, 1872 and *Tonoscolexbirmanicus* within the Megascolecidaeae clade (Fig. 3).

## ﻿Discussion

The mitogenomes of *E.fetida* and *E.andrei* show the same setup as other lumbricid mitogenomes (Boore and Brown 1995; Shekhovtsov and Peltek 2019; Zhang et al. 2019; Shekhovtsov et al. 2020). The nucleotide composition of the mitogenomes was also similar to that of other Lumbricidae species: the AT content of *E.fetida* and *E.andrei* (63.5% and 62.8%, respectively) was comparable to that in Lumbricidae species (59.88–65.69%), including *Eisenianordenskioldi*, *E.balatonica*, *E.tracta*, *E.spelaea*, *Lumbricusterrestris* Linneaus, 1758, and *Aporrectodearosea* (Savigy, 1826) (Shekhovtsov et al. 2020). Zhang et al. (2016) reported higher AT contents in other earthworm families; for example, Megascolecidae has an AT content of 62.6–67.6%, and the Moniligastridae (*Drawidajaponica*) genome has an AT content as high as 69.7%. However, the mitogenomes of *E.fetida* (16,560 bp) and *E.andrei* (15,714 bp) were larger than those of other lumbricid species, such as *L.terrestris* (14,998 bp), *L.rubellus* Hoffmeister, 1845 (15,464 bp), and *Ap.rosea* (15,089 bp). These size differences are primarily due to the extreme length variation of the non-coding region (Shekhovtsov et al. 2020). The length of the non-coding region was 1988 bp in *E.fetida* and 1152 bp in *E.andrei* and significantly longer than those of known mitogenomes of other earthworm species [from 318 bp in *Pontoscolexcorethrurus* (Müller, 1857) to 649 bp in *Metaphirehilgendorfi* (Michaelsen, 1892); Table 1]. In addition, slight differences were observed in the coding regions. The *atp8* gene consists of 54 amino acids in *fetida* and 53 amino acids in *andrei*, whereas *nad5* comprises 567 amino acids in *fetida* and 573 amino acids in *andrei*.

The family Lumbricidae is well-known for its notoriously polyphyletic genera (Domínguez et al. 2015). Unfortunately, only 16 complete or nearly complete Lumbricidae mitogenomes are available in GenBank (including our two new sequences), which prevents us from reaching a comprehensive conclusion on Lumbricidae phylogeny. However, our phylogenetic reconstructions using the available complete or nearly complete mitogenomes corroborated the monophyly of the family Lumbricidae and *Eisenia* (Domínguez et al. 2015; Shekhovtsov et al. 2020), the genus with the most mitogenome sequences (13 sequences) reported, including the type species *Eiseniafetida*. It is interesting to note that the *E.fetida* and *E.andrei* clade along with the Central European *E.spelaea* is distant from the Asian *E.nordenskioldi* species complex, *E.tracta*, and *E.nana*.

Perel (1998) hypothesized that the native range of *E.fetida* is somewhere in the forest-steppe zone of Central Asia, and that the species originally occurred under the bark of fallen logs. In addition, Latif et al. (2017) found surprisingly high morphological and genetic variability of *E.andrei* in northwestern Iran, which demonstrates that the native range of both species is somewhere in western Central Asia. This could explain their closer affinity to the Central European *E.spelaea* than to the Siberian–Far Eastern *E.nordenskioldi* species group.

*Eiseniafetida* and *E.andrei* are sister taxa in both tree topologies (Figs 2, 3), and the branch length between *E.fetida* and *E.andrei* is similar to those of other species on the trees. This supports their distinct species status. However, considering the genetic p-distances of the studied mitogenomes (Table 4), the *E.fetida*/*E.andrei* species pair showed the second smallest genetic distance (14.1%), whereas the p-distance between *L.rubellus* and *L.terrestris* was 18.9% or even larger between the two closely related species *E.nana* and *E.tracta* (19.2%).

**Table 4.** Genetic p-distances of the Lumbricidae mitogenomes.

*Lumbricusterrestris* (U24570)															
*Lumbricusrubellus* (MN102127)	0.189														
*Aporrectodearosea* (NC046733)	0.238	0.231													
*Eiseniafetida* (OK513070)	0.245	0.244	0.223												
*Eiseniaandrei* (OK513069)	0.245	0.243	0.217	0.141											
*Eisenianana* (MK618511)	0.251	0.246	0.231	0.222	0.224										
*Eiseniatracta* (MK642871)	0.245	0.238	0.221	0.209	0.212	0.192									
*Eisenianordenskioldi* (MK618509)	0.260	0.255	0.238	0.233	0.236	0.205	0.204								
*Eisenianordenskioldi* (K618513)	0.246	0.241	0.221	0.213	0.216	0.202	0.179	0.212							
*Eisenianordenskioldi* (MK618510)	0.246	0.24	0.221	0.216	0.217	0.202	0.178	0.213	0.138						
*Eisenianordenskioldi* (MK642867)	0.252	0.249	0.229	0.224	0.225	0.194	0.187	0.206	0.198	0.199					
*Eisenianordenskioldi* (MK642868)	0.257	0.251	0.234	0.226	0.227	0.199	0.196	0.211	0.204	0.199	0.194				
*Eisenianordenskioldi* (MK642869)	0.25	0.245	0.232	0.221	0.22	0.195	0.187	0.204	0.197	0.193	0.191	0.170			
*Eisenianordenskioldi* (MK618512)	0.258	0.25	0.232	0.228	0.232	0.205	0.196	0.218	0.201	0.202	0.196	0.200	0.199		
*Eiseniabalatonica* (MK642872)	0.252	0.248	0.228	0.225	0.224	0.217	0.2	0.225	0.206	0.206	0.209	0.219	0.214	0.220	
*Eiseniaspelaea* (MK642870)	0.264	0.261	0.252	0.221	0.218	0.25	0.245	0.257	0.243	0.242	0.249	0.252	0.251	0.255	0.250

## ﻿Conclusion

On the basis of the mitogenomic analysis of *E.fetida* and *E.andrei*, we can conclude that, although the reproductive isolation between the two taxa is not complete, they should be considered as two independently evolving phylogenetic lineages and, consequently, two separate species.

It is clear that mitogenomes, owing to their highly conserved and highly variable regions, are useful in understanding earthworm systematics at the species and genus/family levels. Addition of other species in future analyses will help to further elucidate the phylogenetic relationships within earthworm families.
